# Three-Dimensional Liver Image Segmentation Using Generative Adversarial Networks Based on Feature Restoration

**DOI:** 10.3389/fmed.2021.794969

**Published:** 2022-01-07

**Authors:** Runnan He, Shiqi Xu, Yashu Liu, Qince Li, Yang Liu, Na Zhao, Yongfeng Yuan, Henggui Zhang

**Affiliations:** ^1^Peng Cheng Laboratory, Shenzhen, China; ^2^School of Computer Science and Technology, Harbin Institute of Technology (HIT), Harbin, China; ^3^School of Instrument Science and Engineering, Southeast University, Nanjing, China; ^4^School of Physics and Astronomy, The University of Manchester, Manchester, United Kingdom; ^5^Key Laboratory of Medical Electrophysiology of Ministry of Education and Medical Electrophysiological Key Laboratory of Sichuan Province, Institute of Cardiovascular Research, Southwest Medical University, Luzhou, China

**Keywords:** CT image, 3D segmentation of liver, semi-supervised, generative adversarial networks, feature restoration

## Abstract

Medical imaging provides a powerful tool for medical diagnosis. In the process of computer-aided diagnosis and treatment of liver cancer based on medical imaging, accurate segmentation of liver region from abdominal CT images is an important step. However, due to defects of liver tissue and limitations of CT imaging procession, the gray level of liver region in CT image is heterogeneous, and the boundary between the liver and those of adjacent tissues and organs is blurred, which makes the liver segmentation an extremely difficult task. In this study, aiming at solving the problem of low segmentation accuracy of the original 3D U-Net network, an improved network based on the three-dimensional (3D) U-Net, is proposed. Moreover, in order to solve the problem of insufficient training data caused by the difficulty of acquiring labeled 3D data, an improved 3D U-Net network is embedded into the framework of generative adversarial networks (GAN), which establishes a semi-supervised 3D liver segmentation optimization algorithm. Finally, considering the problem of poor quality of 3D abdominal fake images generated by utilizing random noise as input, deep convolutional neural networks (DCNN) based on feature restoration method is designed to generate more realistic fake images. By testing the proposed algorithm on the LiTS-2017 and KiTS19 dataset, experimental results show that the proposed semi-supervised 3D liver segmentation method can greatly improve the segmentation performance of liver, with a Dice score of 0.9424 outperforming other methods.

## Introduction

Recent advances in deep convolutional neural networks (DCNN) have shown great promises in handling many computer vision tasks such as target detection, image classification, and semantic segmentation, which can usually reach human-level performance. However, one of the main limitations of DCNN is that they require a large amount of labeled data for training process. This limitation is particularly prominent in dealing with medical image segmentation problems. At present, the acquisition of labeled three-dimensional (3D) medical images requires manual annotation, which is time-consuming and labor-intensive, limiting the further development of DCNN in medical image processing. Moreover, 3D image segmentation for medical applications needs great amount of computing resources, hurdling its practical application. Although the neural network has the characteristics of parameter sharing, it acquires a deeper network structure to improve the performance of the model. As the number of network layers increases, the parameter quantity is increased proportionally. Therefore, the deep neural network needs a large dataset to train the model for obtaining the model parameters. In the absence of sufficient training data, the neural network will have relatively low performance and poor generalization ability.

In addition, there are some problems with liver tissue's structure and CT imaging procession. Firstly, due to the differences in gender, age, and body type of patients, the shape and size of the liver of individual patients are different in appearance in their CT images. Moreover, there are many abdominal organs of compact structure, with tissue density similar to that of liver. Secondly, the area of the diseased area in patients' liver is not fixed in size and with random location that will cause interference to the network in the process of the liver recognition. Finally, there are problems such as sensitivity to noise, metal artifacts, and body motion during the imaging process of CT images, leading to variation of the gray value of liver area due to the influence of the imaging environment, resulting in uneven gray level of liver area, which affects the accuracy of liver segmentation.

Aiming at the difficulty of liver segmentation in abdominal CT images, this paper improves the contrast of the liver in the CT images by preprocessing abdominal CT images, which improves the recognition ability of the liver. Furthermore, semi-supervised learning algorithms reduce the need of large amount of labeled data. In recent years, generative adversarial networks (GAN) have shown great potentials for improving semantic segmentation in a semi-supervised manner (1). Thus, this study also employs GAN to generate fake images by combining labeled CT images to train the network in a semi-supervised manner, which can further improve the algorithm's performance of liver segmentation by expanding the dataset.

The rest of this paper is organized as follows. In section “Related Work”, we give a brief overview of relevant work on liver segmentation. Section “Methods” then presents our 3D liver image segmentation approach based on GAN, which is evaluated and analyzed on the challenging task of liver segmentation in section “Experiments and Discussion”. Finally, we conclude with a summary of our main contributions and results.

## Related Work

Before deep learning was widely used, many methods have been proposed for the liver segmentation of abdominal CT images based on graphics, morphology, and traditional machine learning. With the rapid development of deep learning and its blossom in the field of computer vision, the direction of research in the field of medical image segmentation has also begun to transform to deep learning. In the field of liver image segmentation, more and more methods based on deep learning have also appeared.

### Traditional Methods

Apollon proposed a hybrid liver segmentation algorithm based on pixel intensity threshold (2). It manually selects multiple initial seed points in the image and calculates the average pixel intensity value of nine adjacent pixels of the selected seed points to obtain the segmentation results of the liver image. Amir proposed a two-step liver segmentation method based on threshold and active contour by the contrast characteristics of liver CT image data set, liver shape diversity, and uneven texture (3). Seong proposed an abnormal liver segmentation method based on the adaptive threshold and angle line (4). Moreover, Farzaneh applied the Bayesian-based adaptive threshold to address the issue of liver segmentation (5). This algorithm adjusts the threshold through self-learning to obtain the initial segmentation result of the target area. Then, super pixels are used to constrain the boundary of the liver area for obtaining the final segmentation result. Chen proposed an improved slice-to-slice region growing method, which introduced centroid detection and intensity analysis, and applied morphological operations to extract the liver region (6). Gambino proposed a texture-based volume region growth algorithm, which effectively reduced the impact of artificially selected seed points (7). Lu proposed an improved region growing algorithm for liver segmentation (8). Firstly, the original image is preprocessed by the non-linear mapping. Then, the feature region of the liver is selected through human-computer interaction. Finally, it used the quasi-Monte Carlo method to generate seed points in the feature region, improving the region growth criterion. Rafiei proposed an innovative preprocessing and adaptive 3D region growth method, which uses the map intensity and position of the most probable voxel in the probability map as the region growth boundary to limit the region growth so as to realize the dynamic changes of region growth criterion during the training process (9).

The level set method was first proposed by Osher (10). It has become a classic image segmentation algorithm and has been successfully applied to medical image segmentation problems. Yang proposed a semi-automatic method based on level set and threshold, which includes two level set methods (11). Zhou proposed a liver tumor segmentation algorithm of unified level set by combining regional with boundary information, which is better than applying a single information-driven level set method (12). Alirr proposed a method for automatically segmenting the liver from CT dataset (13). This algorithm utilizes the local shape model and the estimated liver intensity range to establish the initial mask, and then the active contour algorithm is utilized to make the initial mask into the liver boundary. Kass first proposed the active contour model (Snake model) in 1988 (14). This method modeled the problem of image segmentation and transformed it into the problem of minimizing the energy generalization function, which provided a new way of image segmentation. Chi proposed an automatic strategy-based active contour segmentation method for accurate and repeatable liver volume segmentation, which combines rotating template matching, K-means clustering, and local edge enhancement with gradient vector flow model (15). Bereciartua proposed a method for automatic 3D liver segmentation, which achieved liver segmentation by minimizing the fully variable dual (16).

Chen proposed a two-step liver segmentation method based on low-contrast images (17). In the first step, K-Means clustering algorithm and prior knowledge are applied to find and identify liver and non-liver pixels. In the second step, the liver is segmented from the low-contrast image based on graph cutting. Sangewar proposed a new variational model for segmentation of liver regions based on the idea of intensity probability distribution and regional appearance propagation, which overcomes the poor segmentation results caused by the low contrast and edge blur of liver CT images (18).

### Deep Learning Methods

Deep learning (DL), as a branch of machine learning, has shown potentials in medical image segmentation (19). When using deep convolutional neural networks for organ segmentation, thanks to its powerful feature extraction capabilities, it can accurately extract the complex and semantically rich feature information of organs, making the network have high segmentation capabilities (20). The advantages of deep learning are incomparable to traditional machine learning algorithms. Therefore, the current mainstream segmentation algorithms are based on deep learning, and the segmentation accuracy is generally better than traditional segmentation algorithms.

There are various types of segmentation methods. Some researchers applied two-dimensional (2D) convolutional neural networks (CNN) to deal with liver segmentation by learning 2D context of the image (21, 22). Others designed models with 3D contexts only in small voxels due to the high computation cost and memory consumption of 3D CNN (23–25). Furthermore, they used several 2D CNNs that are combined to enhance 2D contexts during the liver segmentation (26, 27). Finally, the 2D and 3D contexts were considered to fuse for training the network (28–31).

For the research of live segmentation, Ben-Cohen directly applied the full convolutional network on a relatively small liver dataset for liver and lesion segmentation. However, the segmentation results were not ideal (32). Christ proposed a way to automatically segment liver and lesions in CT abdominal images using cascaded fully convolutional networks and dense 3D conditional random fields for the joint segmentation of the liver and its lesions to achieve ideal effect (33). Yao proposed a cascade structure to realize automatic segmentation of liver CT images (34). A fully convolutional network is trained to roughly segment the liver, and then the conditional random field model is used as a post-processing refinement liver segmentation to improve the effect.

## Methods

### Overview of the Framework

**Figure 1:**
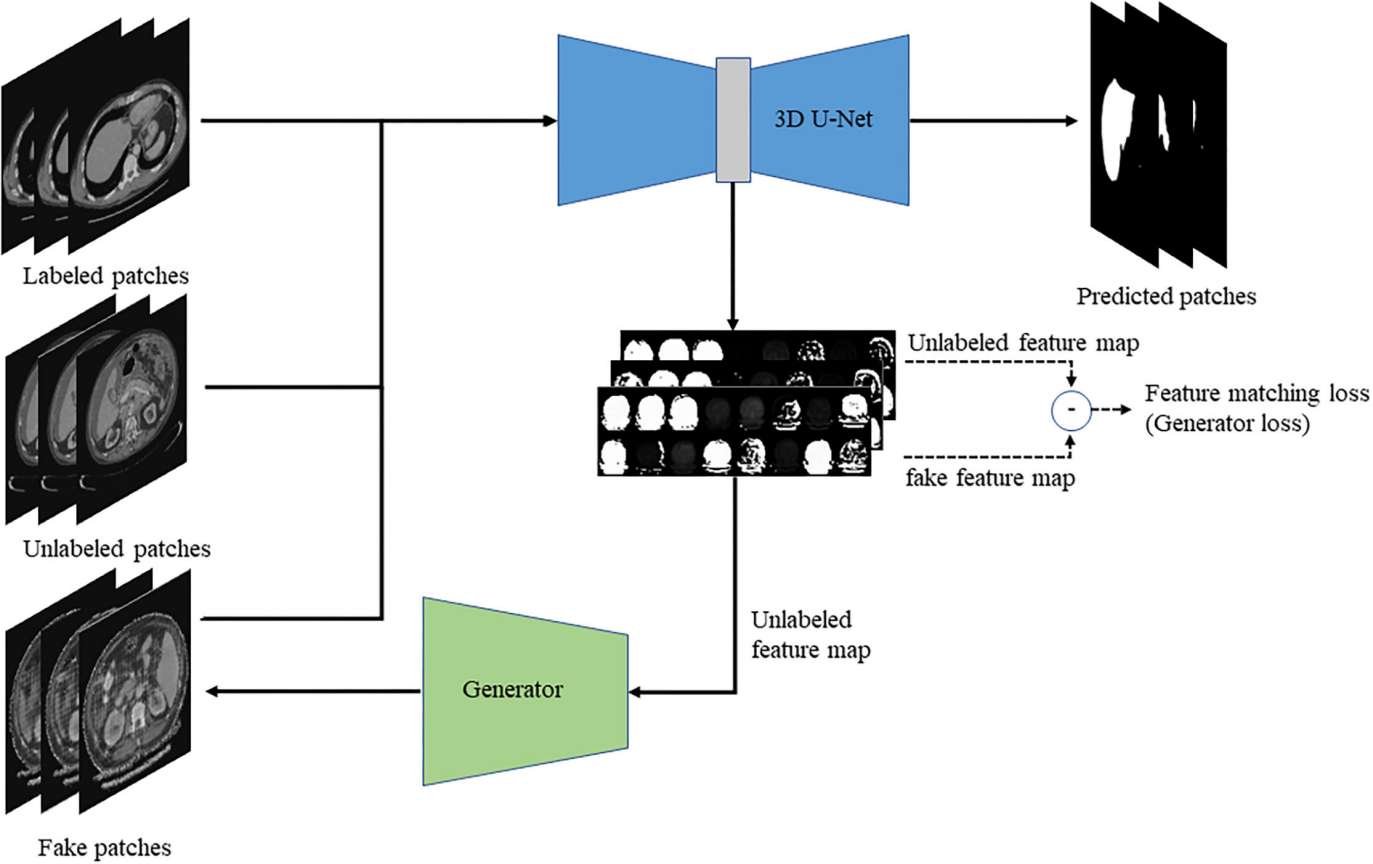
The schematic diagram of the semi-supervised deep learning framework for liver segmentation.

This paper proposed a semi-supervised 3D liver segmentation method based on deep convolutional GAN (DCGAN), which consists of the discriminator and generator. Among them, the improved 3D U-Net network is applied as a discriminator to identify real images and generated fake images and obtain the 3D segmentation results of the liver. Then, we design a DCNN based on feature restoration method to generate fake images by the feature map of the real images. The network structure of the optimization segmentation algorithm based on the GAN is shown in Figure 1.

### Preprocessing

Data preprocessing is an important means to improve the effect of deep learning training, which can adjust the overall distribution of the sample to make it more suitable for training. Improving the quality of the sample can make the model easier to fit the feature distribution of the sample. In CT images, CT value is used to measure the density of human tissues or organs by the Hounsfield Unit (HU). The CT value range is generally [−1,000HU, +1,000HU], in which air is −1,000HU and dense bone is +1,000HU. Therefore, the CT value in the image needs to be converted into gray value before the liver segmentation. The main steps are as follows:

(1) CT value truncation: As the highest contrast range of the liver in the images is [−200HU, +200HU], so we cut the CT value to a certain range, which the CT values smaller and larger than −200HU and +200HU are set to −200HU and +200HU to accomplish the CT value truncation. Clipping is used to improve the contrast between the liver and other tissues. This is a key step in preprocessing CT images. Without clipping, the segmentation performance of CT images is poor, and after the clipping processing, the network can also converge faster. In addition, this paper is mainly to segment the liver, which is not sensitive to bony structure information. By clipping the intensity range of CT images, the interference of bones and other tissues can be reduced.
(2) CT value normalization: CT images in the dataset were obtained from several image acquisition sources with various scanning equipment and imaging environments, which led to different imaging effects and grayscales. Such difference in the gray level has a greater impact on the training process of the samples. Therefore, it is necessary to eliminate the influence of imaging differences as much as possible in the process of converting the CT value to the gray value. T The normalized formula is shown in Formula (1):

(1) $$\begin{array}{r} H^{\prime }\left(x,\;y,z\right)=\frac{H\left(x,\;y,z\right)-{HU}_{\min}}{{HU}_{\max}-{HU}_{\min}}, \end{array}$$

where the values of *HU*_max_ and *HU*_min_ are +200 and −200, *H*(*x, y, z*) represents the CT value of the voxel with coordinates (*x, y, z*) in the CT image before normalization, and *H*′(*x, y, z*) represents the normalized value.

(3) Gray value interval mapping: The normalization is set to facilitate the calculation and supervision of the training process. In the normalization stage, we multiply the normalized value by 255 according to the range of RGB value and convert it to an integer. Moreover, the normalized value below zero is invisible to the naked eye, therefore, it needs to be multiplied by a reasonable value in order to make the contrast of the image clearer. Thereby the CT value of [−200, +200] is mapped to the gray value interval of [0, 255].

### Improved 3D U-Net Network Structure

The U-Net model was originally designed to solve the task of 2D medical image segmentation, which all network layers in the model are 2D. In order to realize the 3D segmentation of the images, a 3D version of the U-Net model needs to be applied. On the basis of not changing the original encode-decode structure of model, all the network layers in the model are replaced with a 3D type to obtain the 3D U-Net segmentation model (35). The 3D U-Net network structure is used to segment 3D images through the extension of the classic U-Net network in processing data dimensions. Compared with the classic U-Net network structure, in addition to the difference in the dimension of the convolution kernel, the 3D U-Net network only performs three down-sampling operations followed by one batch normalization (BN) layer. In this paper, the liver 3D segmentation algorithm is to increase the performance of the network by adding some modules on the basis of the 3D U-Net network. The specific improvements and operations are described as follows.

#### Squeeze and Excitation (SE) Module

**Figure 2:**
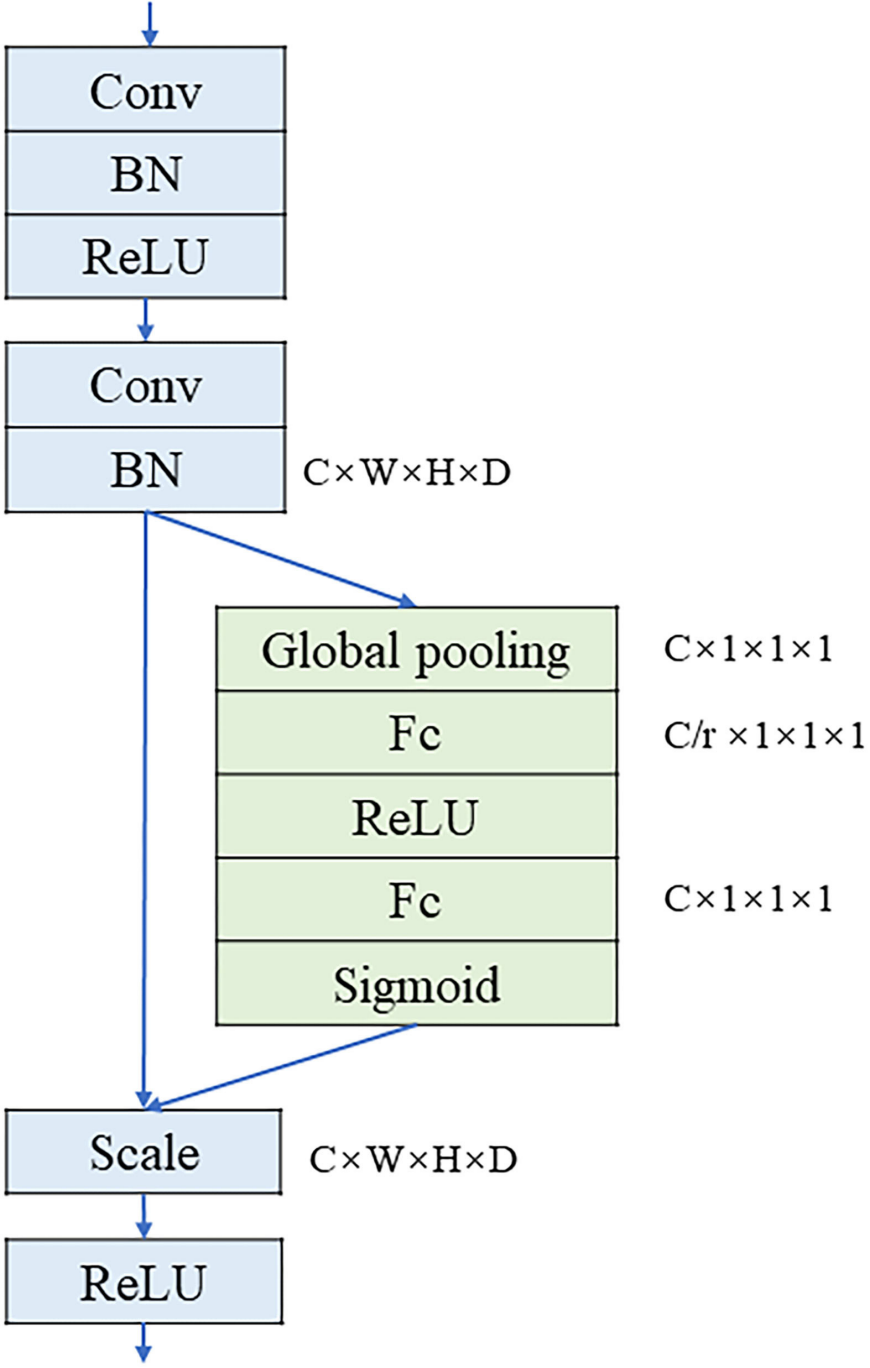
The structure of SE module.

The original 3D U-Net model only uses convolution to extract features. This paper has added SE structure to extract image features, which can weight each feature channel according to the value of the feature image to increase the weight of important features and reduce the weight of the irrelevant features, thereby improving the effect of feature extraction. The SE structure is an attention mechanism based on feature channel weighting (36). In this paper, the 3D SE structure and convolutional layer are combined as the basic convolution module, which is called the SE module. The SE module consists of two convolutional layers. The first convolutional layer adjusts the resolution and the number of channels of the input feature map to a specific size. In addition, it can compress the feature channel to reduce the amount of calculation. The second convolutional layer is utilized in conjunction with the SE structure. The SE structure first performs global pooling on the feature map and applies the bottleneck structure to finally obtain the weights of each channel with a value range [0, 1] by the sigmoid activation function. The structure of the SE module is shown in Figure 2.

Among them, W × H × D × C represents the size of the feature map, W, H, and D represent the width, height, and depth of the feature map, respectively, C represents the number of channels of the feature map, and r is the multiple of the restoration of the number of channels in the Excitation operation. Scale is a weighted operation which is followed by the rectified linear unit (ReLU) activation function to output the result.

#### Pyramid Pooling Module

The 3D U-Net model only uses three downsamplings to obtain the receptive field of the 3D image, and it elevates the role of shallow features by jump connections allowing the model to determine the importance of different scale receptive fields. In this study, we have introduced the pyramid pooling module to obtain a larger receptive field (37). The pyramid pooling module applies multiple scales of pooling operations to obtain and fuse feature information of multiple scales, which can improve the degree of freedom of the model for multi-scale receptive field selection. It also can add multi-scale information of features without affecting the original features. Furthermore, in order to splice the original feature image with the pooling results of different scales, it is necessary to make the pooling results of different scales to be the same size as the original feature map. This module enables each location to obtain the information of multiple ranges. Thus, the maximum range can directly reach the global size, and the module can quickly acquire a larger variety of information. This article will use the 3D structure of the module to obtain a larger range of receptive fields. Firstly, we applied the 1 × 1 convolution operation to perform feature channel fusion for each scale pooling result, and it is up-sampled to the original feature map size and spliced. Then, the number of feature channels is also reduced through the 1 × 1 convolution operation, and finally spliced with the original feature map as the output.

#### Improved 3D U-Net Model

**Figure 3:**
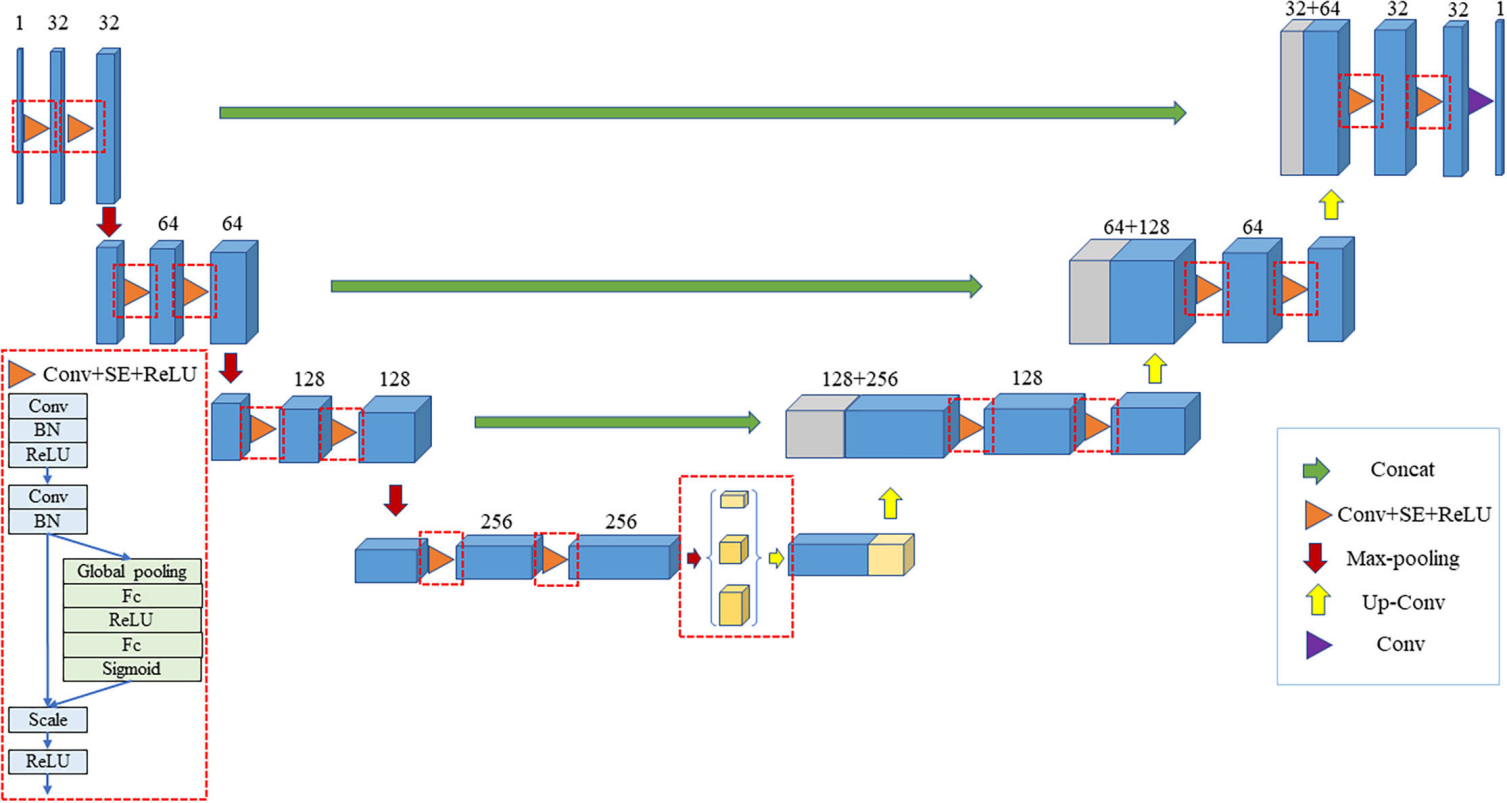
Improved 3D U-Net model.

In order to improve the segmentation accuracy of the original 3D U-Net network, an improved network based on 3D U-Net is proposed to perform the 3D segmentation of the liver. Firstly, all the convolutional layers in the original 3D U-Net network are replaced with the SE module. The improved structure is a deep learning model based on the encoder-decoder structure, in which the encoder is composed of SE module and down-sampling, which is mainly responsible for extracting features and expanding the receptive field. The decoder consists of SE module and up-sampling, which has the main function of extracting features and expanding resolution. There is a skip connection between the encoder and the decoder. The skip connection splices the shallow features in the encoder with the deep features in the decoder, and the shallow features provide detailed information for the decoder. The purpose is to make the network pay more attention to the feature information related to the liver and increase its importance while reducing the role of irrelevant information such as background, enabling it to obtain a finer segmentation boundary. In addition, the pyramid pooling module was introduced to make the network obtain multi-scale feature information and expand the receptive field of network. In the improved 3D U-Net network, the pyramid pooling module is added at the end of the encoding path that has the smallest resolution in the entire network. The modified network structure is shown in Figure 3.

The encoding part is shown in the left half of the figure. In the encoding part, the model applies four SE module groups of which each module group consists of two SE modules. The first convolution step in the first SE module is 2 while the number of channels is increased. After the four SE module groups, we have added the pyramid pooling module that contains 3 parallel average pooling layers with sizes of 1, 2, and 5, respectively. The encoding part includes a total of 4 down-sampling, which are all performed by convolution with a step size of 2. The pyramid pooling module is added to the end of the encoding path that is the place with the smallest resolution in the entire network.

The decoding part is shown in the right half of the figure. The first part of decoding consists of three SE module groups—each of which contains two SE modules. The first SE module has two inputs: one is the output from the previous layer of the decoding part, and the other is the output from the corresponding position of the encoding part. The module first applies the transposed convolution with a step size of 2 to expand the output resolution of the previous layer to double the original resolution, reduce the number of channels to 1/2 of the original, and splice the output at the corresponding position of the encoding part. After that, it performs another SE convolution operation. After three SE module groups, the feature image is restored to the original image size using the transposed convolution. After splicing with the original image, it obtains the final segmentation result.

### Deep Convolutional Generative Adversarial Networks (DCGAN)

At present, convolutional neural networks have been widely applied in generative adversarial networks. However, generative adversarial networks lacked a general network architecture until the emergence of DCGAN, which is an unsupervised learning algorithm combining deep convolutional neural networks and generative adversarial networks (38). The design idea of DCGAN is to restrict the network structure based on the original network framework to achieve a more powerful generative model.

The generator of DCGAN generates fake images by convolution and up-sampling of random noise, which is widely used in the task of generating 2D images. To generate a 3D image, the effect of using random noise is very poor because of the difficulty to learn the 3D image distribution through a deep neural network. Learning high-dimensional image distribution is very slow by using noise as input, while using 3D U-Net network as a discriminator is very quick to converge during the training process. The contradiction will cause the problem of gradient dispersion. In addition, we have used random noise to generate CT images. However, the contours of the CT images are difficult to generate, let alone the internal distribution. In order to generate more realistic 3D images, it is necessary to add more real image distribution information to replace random noise, which can speed up the learning rate of the generator.

**Figure 4:**
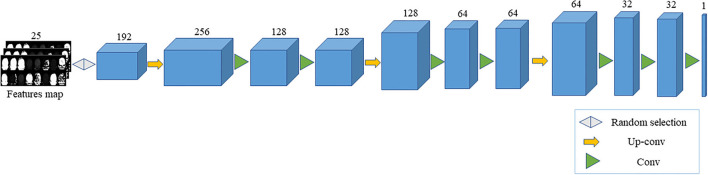
The generator structure.

In this article, we design a convolutional neural network based on feature restoration method. By extracting the feature map generated by the improved 3D U-Net network, a part of the feature map is randomly selected as the input of neural network due to the following reasons. Recovering all the feature maps is a reverse process of feature extraction, which the distribution obtained is the same as the real image. If a part of the feature map is selected, the generator will learn the real image distribution and complete the missing parts. Moreover, the image obtained is different from the real image which increases the variety of images and achieves the purpose of expanding the dataset. Because the feature map is randomly selected, the missing part is also random, and, therefore, the generator can be trained to restore the real image at any position. In addition, the reason why the feature map, rather than the partially missing real image, is applied is that the real image contains much useless information, which causes the generator to converge slowly. Through up-sampling and convolution operations, a fake image with the same size as the real image slice is obtained. After that, the feature map of fake and real images are, respectively, extracted through the improved 3D U-Net network, and the mean difference between the two feature maps is applied as the loss. Then, the network parameters of generator are updated through multiple iterations, making the generator better restore the feature map and, also, making a fake image closer to the real image. The generator structure is shown in Figure 4.

### The Definition of Label and Loss Function

The proposed segmentation optimization algorithm is semi-supervised. There are fully supervised learning for labeled data and unsupervised learning for unlabeled data and fake images. Therefore, the labels need to be redefined so that the discriminator can identify fake images. The original label defines the background and liver as 0 and 1. Now a new label category needs to be added to mark the fake images, in which the label is defined as 2. The output size of the improved 3D U-Net network is H × W × D × 3, 3 represents the number of labels, the output vector of each voxel is [*l*_i, 1_, *l*_i, 2_, *l*_i, 3_], which represents the probability that the current voxel is false. In order to learn from unlabeled data, its output is forced to be a label of the real data that is achieved by maximizing the output vector.

The loss function of the discriminator is shown in Formula (2):


(2) $$\begin{array}{r} L_{discriminator}\;{=\;L}_{labeled}+L_{unlabeled}\;+\;L_{fake} \end{array}$$

where *L*_*discriminator*_ is the loss of the discriminator, *L*_*labeled*_ is the loss of labeled data, *L*_*unlabeled*_ is the loss of unlabeled data, and *L*_*fake*_ is the loss of fake images generated by the generator.

For labeled data, we use cross entropy loss function to calculate as shown in Formula (3):


(3) $$\begin{array}{r} L_{labeled}\;=\;-E_{x,y\sim p_{data}\left(x,y\right)}\overset{H\times W\times D}{\underset{i=1}{\sum }}\log P_{model}\left(y_{i}|x,y_{i}<K+1\right) \end{array}$$

where *x* represents the input image, *K* represents the number of classes labels, *y* represents labeled image, *x, y*~*p*_*data*_(*x, y*) represents that the input image is labeled, and *p*_*model*_(*y*_*i*_*|x, y*_*i*_<*K*+1) represents the probability of the voxel prediction category is *y*_*i*_ in the image.

For unlabeled data, the loss function is shown in Formula (4):


(4) $$\begin{array}{r} L_{unlabeled}\;=\;-E_{x\sim p_{data}\left(x,y\right)}\overset{H\times W\times D}{\underset{i\;=\;1}{\sum }}\log\left({1-P}_{model}\left(y_{i}|x,y_{i}<K+1\right)\right) \end{array}$$

where x represents the input image, *x*~*p*_*data*_(*x, y*) represents that the input image is unlabeled, and *p*_*model*_(*y*_*i*_*|x, y*_*i*_<*K*+1) represents the probability of the voxel prediction category is fake images.

For the fake images, the loss function is shown in Formula (5):


(5) $$L_{fake}\;=\;-E_{e\sim Encoder\left(x\right)}\sum_{i=1}^{H\times W\times D}\log P_{model}\left(y_{i}\;=\;K+1|G_{\theta_{G}}\left(e\right)\right)$$

where *x* represents the input unlabeled image, *e*~*Encoder* (*x*) represents the fake images generated by the generator based on the unlabeled image distribution, and *p*_model_ (*y*_*i*_=*K*+1|*G*_θ*G*_(*e*)) represents the probability that the voxel prediction category is a fake image in the fake images.

The loss function of generator is shown in Formula (6):


(6) $$\begin{array}{r} L_{generator}\;=\;||E_{e\sim Encoder\left(x\right)}f\left(x\right)-E_{e\sim Encoder\left(x\right)}f\left(G_{\theta_{G}}\left(e\right)\right)|{|}_{2}^{2} \end{array}$$

where x represents the input unlabeled image, *f* (*x*) represents the extracted feature map from the unlabeled images through the 3D U-Net network decoder, *e* represents the fake image generated by the generator, *f* (*G*_θ*G*_(*e*)) represents the extracted feature map from the fake images through the 3D U-Net network decoder.

## Experiments and Discussion

### Datasets

LiTS-2017 is a liver tumor segmentation challenge dataset launched in 2017 (39). The data set includes 100 3D abdominal CT scan images (nii format). In the experiment, 60 images were selected as the training set, and the validation and test set contain 20 images, respectively. The training set is divided into 50 labeled and 10 unlabeled images. The labeled and unlabeled images are applied to train the segmentation network and generate fake images. Firstly, we scale each image in the dataset and intercept the liver position to change the size of the image to 256 × 256 × *N*. Then, the image is cut into patches with the size of 256 × 256 × 16 to obtain a total of 500 patches for training, which the number of labeled and unlabeled patches is 400 and 100. In the meantime, the validation and test set are processed to contain 200 patches, respectively. Moreover, in order to verify the generalization of the proposed model, we applied another dataset, named KiTS19, which is a kidney tumor segmentation challenge dataset launched in 2019 (40). The dataset includes 200 3D abdominal CT scan images (nii format). For the dataset, we perform the same operations as the LiTS-2017. Finally, a total of 1,000 patches are generated for training, of which the number of labeled and unlabeled patches is 900 and 100. In the meantime, the validation and test set are processed to contain 210patches, respectively.

### Evaluation Etrics

To evaluate the segmentation performance of the proposed network, we adopted the widely used segmentation evaluation metric: Dice coefficient (Dice) (41). Dice is a function of ensemble similarity measurement to calculate the similarity of two samples with the range [0, 1] at the pixel level. The real target (Ground truth) appears in a certain area A, and the target area of the model prediction result is B. Then the calculation of Dice is shown in formula (7):


(7) $$\begin{array}{r} Dice\left(A,B\right)=\frac{2|A\cap B|}{A+B} \end{array}$$

where |*A*n*B*| represents the intersection between A and B, |*A*| and |*B*| represent the total number of *A* and *B* pixels, respectively. Because there are overlapping elements between A and B in the denominator *A* + *B*, it adds a coefficient 2 to the numerator. In the problem of medical image segmentation, *A* and *B* represent the real label image and the segmented image predicted by the model.

### Training Process

#### Generation of Fake Images

**Figure 5:**
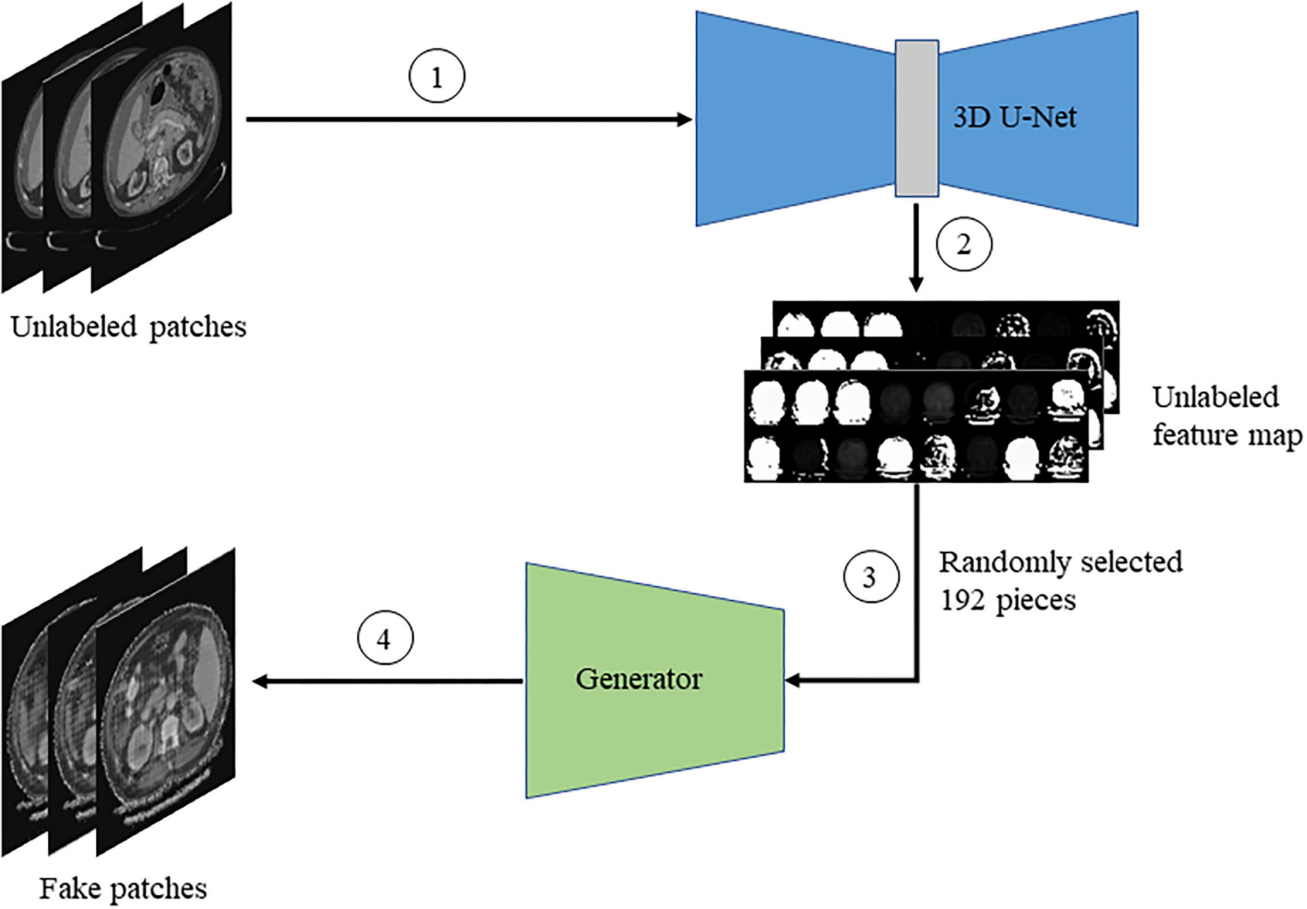
The flow chart of generating fake images.

Firstly, the unlabeled image is input into the improved 3D U-Net network to obtain the feature map. Then, we randomly select a part of the feature map as the input of the generator. Finally, the generator generates fake images base on feature restoration method. The flowchart is shown in Figure 5.

#### Discriminator Training

**Figure 6:**
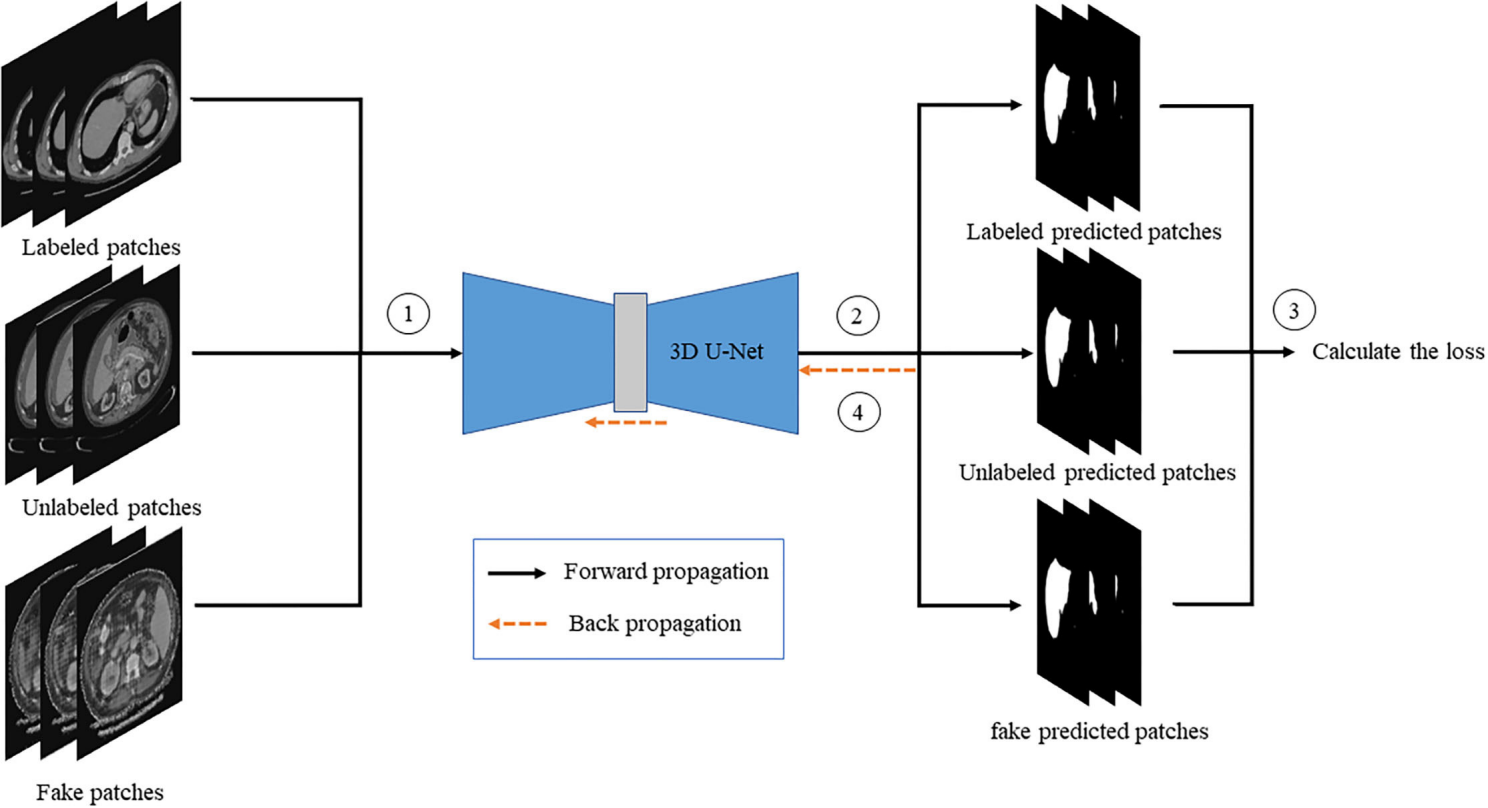
The flow chart of discriminator training.

The labeled, unlabeled, and fake images are, respectively, passed into the discriminator, and then the loss of the discriminator is calculated to update the gradient of the discriminator. The flowchart is shown in Figure 6.

#### Generator Training

**Figure 7:**
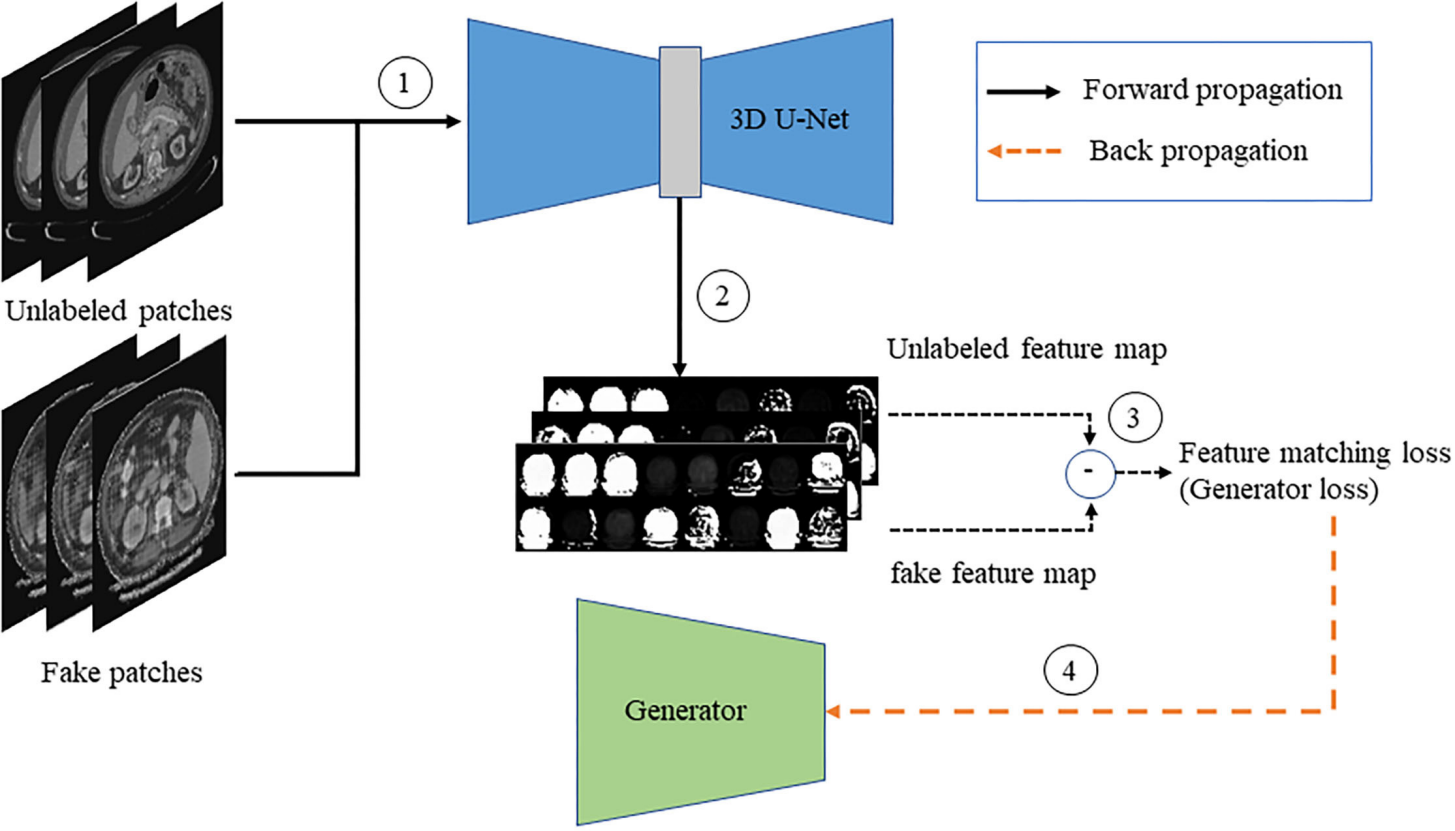
The flow chart of generator training.

The fake and unlabeled images are, respectively, passed to the improved 3D U-Net network after the gradient update to obtain the feature map, and the generator loss is calculated according to the similarity of the feature map to update the gradient of the generator. The flowchart is shown in Figure 7.

### The Results of Generated Images

**Figure 8:**
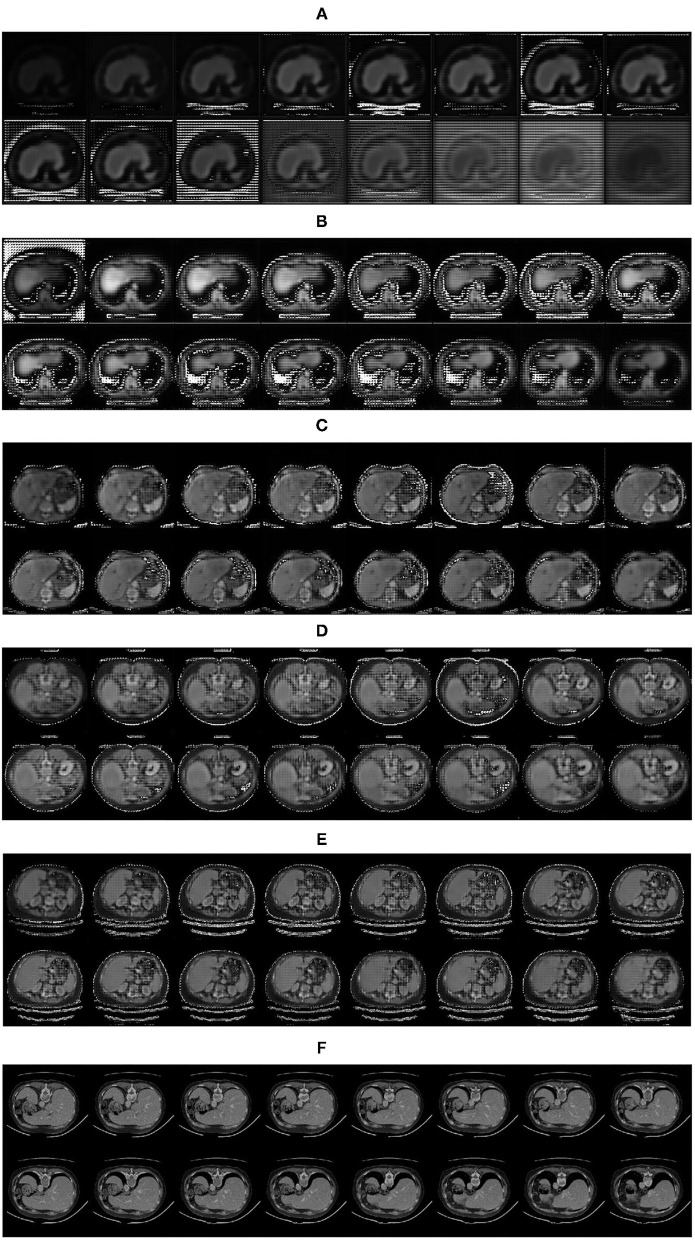
Fake images generated by the generator: **(A)** 1,000 iterations; **(B)** 5,000 iterations; **(C)** 10,000 iterations; **(D)** 15,000 iterations; **(E)** 20,000 iterations; **(F)** Real images.

The experiment proves that the fake image is very close to the real image by the feature restoration method, and the speed of the network learning distribution is faster than that of the network using random noise as the input, which avoids the problem of gradient dispersion. The fake images generated by the generator is shown in Figure 8.

### Experimental Results

#### Comparison of Experimental Results

For each group of experiments, the segmentation results were verified on the same public dataset LiTS-2017. In the process of network training, the Adam optimization algorithm is applied, the learning rate of discriminator and generator are set to 10^−4^ and 3 × 10^−4^, and the batch size is set to 1. We have trained the original 3D U-Net network, the improved 3D U-Net network, and the generative adversarial network based on the feature restoration method, respectively, and set up comparative experiments for the improvement of each part.

In this study, each ablation experiment uses the same training set, verification set and the test set, of which results are shown in Table 1. From the comparison experiment, it can be seen that the improved 3D U-Net network has improved compared with the original 3D U-Net network in the validation set. The improved 3D U-Net network also has a certain improvement over the original 3D U-Net in the test, especially the introduction of the GAN. The addition of the GAN not only improves the segmentation effect of the training set and validation set, but also improves the performance of the test set. For the improvement of segmentation performance, we have analyzed the contribution of each module, which are presented as follows.

**Table 1:** Comparison of experimental results on the LiTS-2017 dataset.

**Method**	**Validation set**	**Test set**
	**dice coefficient**	**dice coefficient**
3D U-Net	0.9160	0.881
3D U-Net+SE+Pyramid pooling	0.9304	0.905
3D U-Net+SE+Pyramid pooling+GAN	**0.9638**	**0.942**

*The bold values represent the highest score*.

Similar to the attention mechanism, the SE module integrated in the original 3D U-Net network extracts the relationship between the channels using the global information of each channel and weights each channel. The SE module can determine the importance of various features according to the value of each channel, which can effectively improve the performance of the model. Adding the pyramid pooling module, the application of multi-scale pooling layer allows the model to obtain different sizes of receptive fields, while further expanding the receptive field of model. The two operations, together, improve the feature extraction capability of the model. Furthermore, the GAN, based on the feature restoration method, can generate more diverse images containing real distribution information. On a limited dataset, it can improve the segmentation performance and generalization of the model, which is a promising way for the generation of 3D medical images.

Finally, we have compared the proposed algorithm with others. As shown in Table 2, our algorithm is better than the DenseNet and 3D DenseUNet-65 algorithms (42, 43). In addition, the performance of the proposed method is slightly lower than the FCN+ACM method (44). The GIU-Net algorithm (45) outperformed our proposed model for liver segmentation. A suitable explanation for that is that they utilized more data to train the model, while our model is trained on a limited dataset. In the future, we will further improve the performance and reduce the complexity of the model.

**Table 2:** Comparison of experimental results with other methods.

**Method**	**Dice coefficient**
DenseNet (42)	0.923
3D DenseUNet-65 (43)	0.929
FCN+ACM (44)	0.943
GIU-Net (45)	**0.951**
3D U-Net+SE+Pyramid pooling+GAN	0.942

*The bold values represent the highest score*.

#### The Segmentation Results

**Figure 9:**
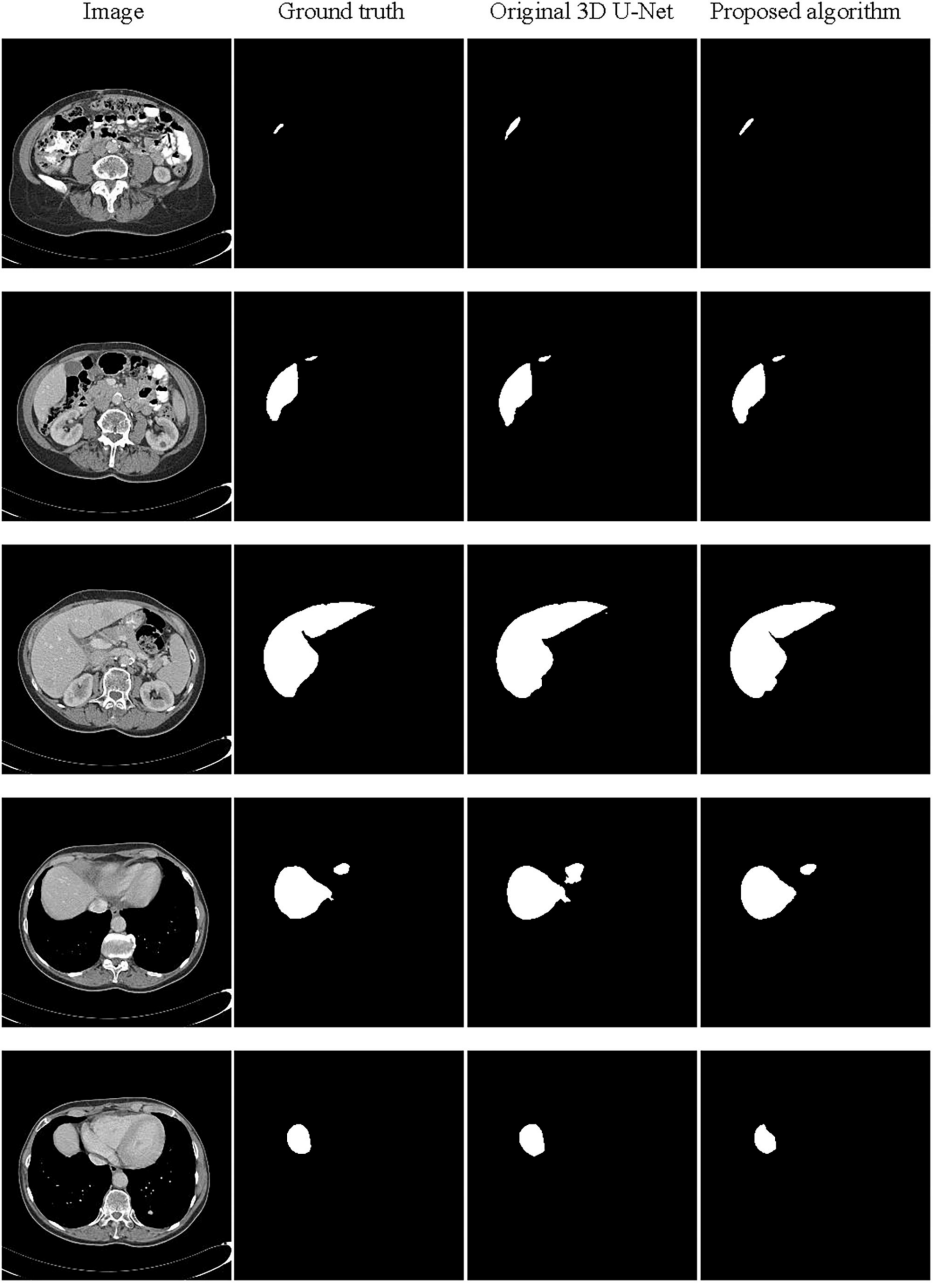
Schematic representation of the liver 3D segmentation results.

The partial segmentation result is selected from the test set as shown in Figure 9. The first column is the original image, the second column is the ground truth, and the third and last column are the segmentation results by the original 3D U-Net and proposed algorithm. It can be found that using the proposed algorithm, the 3D segmentation results of the liver are very close to the real labels, which the problem of poor segmentation accuracy of head and tail regions of the liver has been improved. In order to further analyze the segmentation results, we have also supplied the 3D surface plots with the color bar of the Hausdorff Distance (HD).

**Figure 10:**
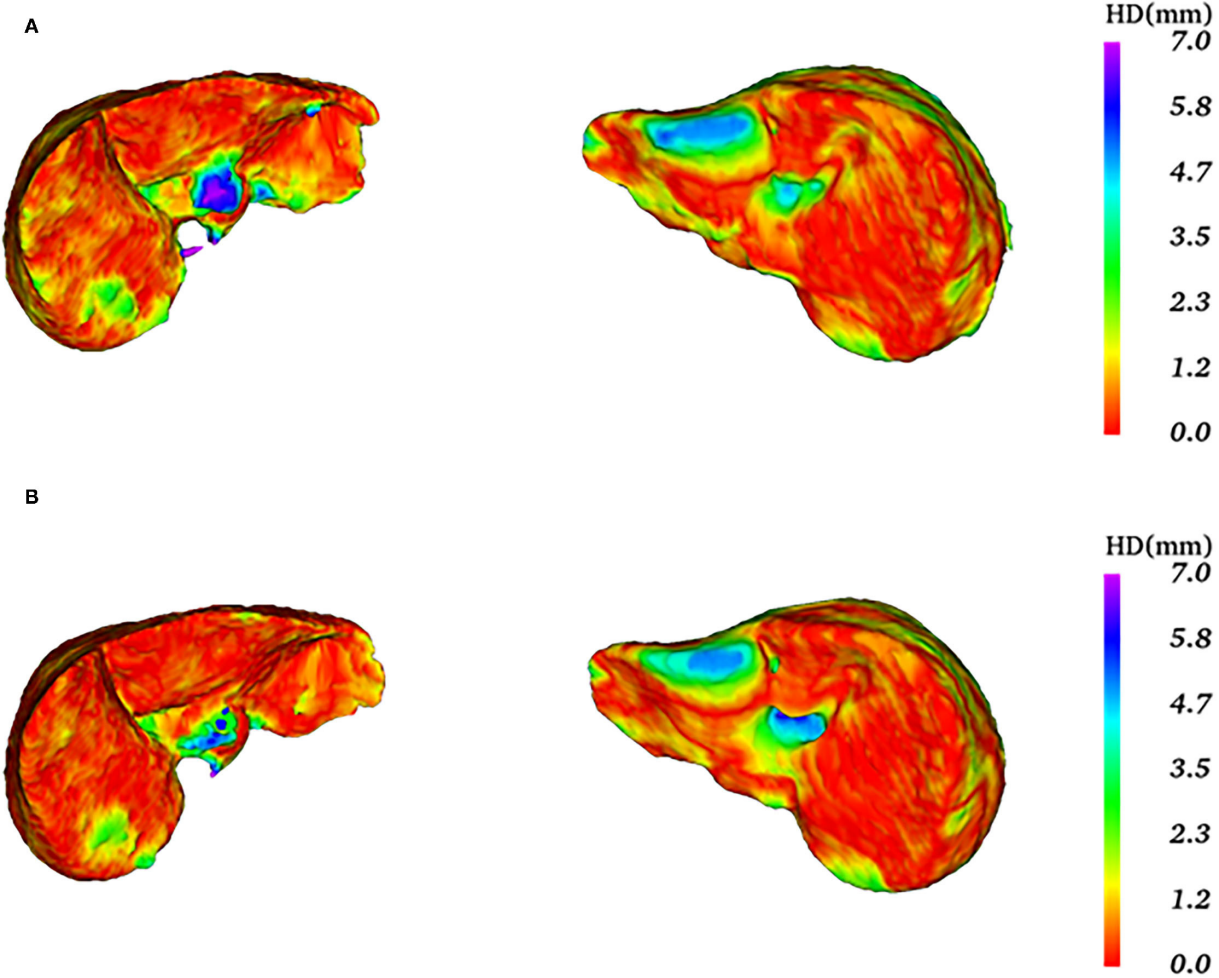
Comparison of the 3D surface plots of the two algorithms.

In Figure 10, the first row is the 3D surface plot generated by the original 3D U-Net network for two representative test data, while the second row is generated by the proposed algorithm. As we can see in the left column, the HD values of these two algorithms are relatively high. The 3D surface plot above, generated by the original 3D U-Net network, has a redundant prediction (the blue area) and obtains an HD value of 18.68 mm. The 3D surface plot below is generated by the proposed algorithm that has some outliers with the purple area, which has a greater impact on HD obtaining the HD value of 15.33 mm. In addition, the average symmetric surface distance (ASSD) values are 0.93 and 0.64 mm for the original 3D U-Net network and proposed algorithm, respectively. For this test data, the segmentation results of the two algorithms are not very good. However, the proposed algorithm still performs better than the original 3D U-Net network. For the right column, the segmentation effect of the two algorithms is better than the previous one. The 3D surface plot above was generated by the original 3D U-Net network whose boundary segmentation is not ideal. In this case, the HD value of 9.70 mm. The 3D surface plot below is generated by the proposed algorithm that is better than that the original 3D U-Net network and reduces the HD value (8.06 mm). Moreover, the average symmetric surface distance (ASSD) value of the original 3D U-Net network and proposed algorithm are 0.93 and 0.87 mm. For the scores of HD and ASSD, they are not as ideal as the Dice coefficient, because that we use the Dice coefficient to determine the end of training and the model is more inclined to calculate Dice to a certain degree. In the future, we will further improve the performance of the model.

### Evaluation of Model Generalization

Generally speaking, a network model is proposed to solve and optimize a certain problem for obtaining satisfactory results. Therefore, most of models have poor generalization ability and can only be applied to a certain area or dataset. In order to establish a general network model, the accuracy may be sacrificed. However, thanks to the powerful generation ability of the generative adversarial network, the distribution of different organs can be learned through iteration to generate fake images. By this expansion of the dataset, the generalization ability of the model can be greatly improved. In this study, the proposed 3D liver segmentation model is applied to the KiTS19 kidney dataset to test the generalization ability of the model. The results are shown in Table 3.

**Table 3:** Comparison of experimental results on the KiTS19 dataset.

**Method**	**Validation set**	**Test set**
	**dice coefficient**	**dice coefficient**
3D-UNet	0.906	0.871
3D-UNet+SE+Pyramid pooling+GAN	**0.959**	**0.959**

*The bold values represent the highest score*.

It can be seen from the experimental results that the model still performs well for the 3D segmentation of the kidney. Comparing with the classic 3D U-Net network, the accuracy of kidney segmentation is significantly improved on the verification set. Furthermore, the segmentation ability of the model is still strong on the test set, even exceeding the performance of the verification set.

**Figure 11:**
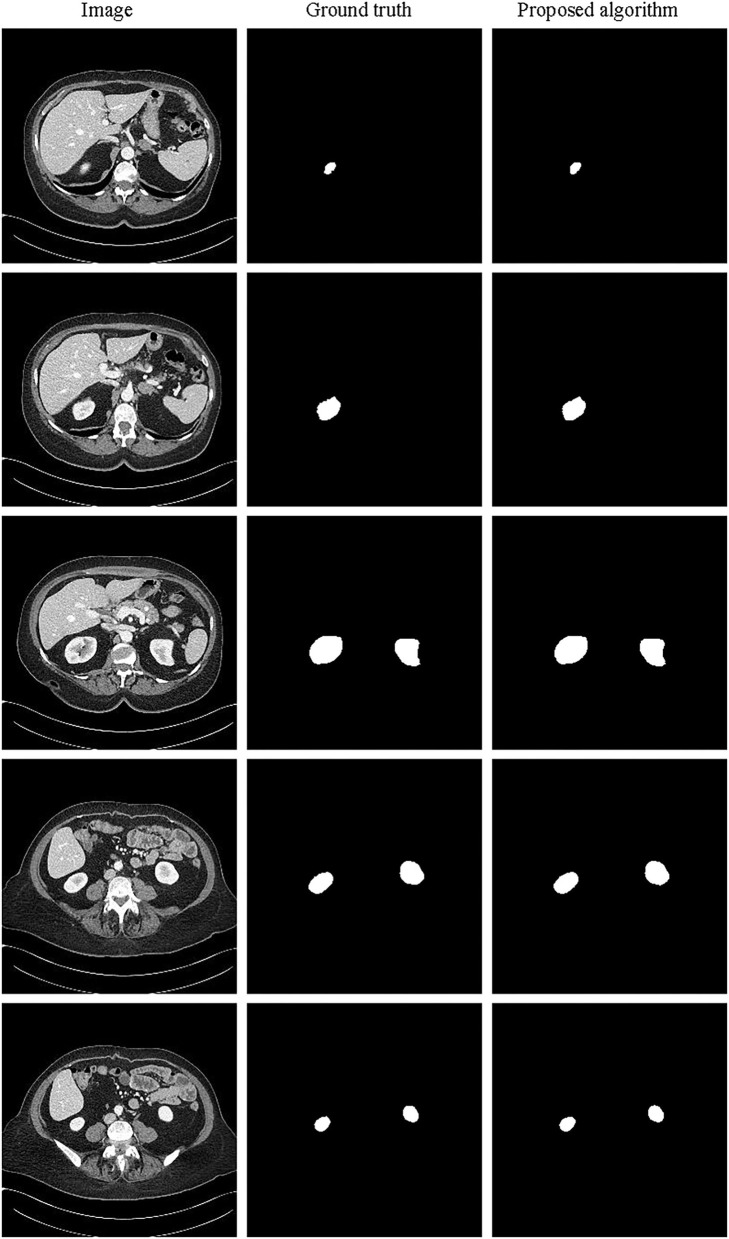
Schematic representation of the kidney 3D segmentation results.

The partial segmentation result is selected from the test set as shown in Figure 11. The left column is the original image, the middle column is the annotated kidney image, and the right column is the segmentation result. It can be found that the 3D segmentation results of the kidney are also very close to the real labels. Since it is only to verify the generalization of the image generation method based on feature restoration, it has not been further analyzed. In the future, we will further study and verify the general 3D image generation method.

### The Contribution and Future Work

Aiming at the problem of low segmentation accuracy of the original 3D U-Net network, an improved network based on 3D U-Net is proposed to perform the 3D segmentation of the liver. In order to make the network pay more attention to the characteristic information of the liver and reduce the role of irrelevant information, such as background, we introduce the squeeze and excitation (SE) module to the network. Meanwhile, in order to allow the network to obtain feature information of multiple scales and expand the receptive field of the network, we also introduce the pyramid pooling module to the model. Through the combination of the two modules, we have improved the overall segmentation performance of the liver.

In view of the lack of labeled 3D data, we embed the improved 3D U-Net network in the GAN as the discriminator and propose a semi-supervised liver segmentation method. The limited labeled images and unlabeled images are used to train the learning model to generate fake images for expanding the dataset. Aiming at the poor quality of generating 3D abdominal fake images by using random noise as input, a DCNN based on feature restoration method is designed to generate more realistic fake images using randomly selected feature maps, which is embedded in the GAN as the generator. Based on the feature restoration method, the generator can make better use of the real image distribution information to generate more realistic images, which increases the diversity of the images.

However, the network has a large number of parameters, which leads to a long training period when the computing resources are limited. Because the data is 3D volume of the liver, the encoder-decoder structure is applied to extract features and restore 3D images every time during the training. In addition, the generator needs to use the random feature map generated by the encoding part of the improved 3D U-Net model to generate fake images. Therefore, it takes up massive video memory during the training of the model. In this study, the training of the proposed model (100 epochs) required approximately 50 h on a single NVIDIA GTX 1080Ti with 11 GB, indicating that each epoch takes about 30 min. The total parameters of the proposed network are about 150 million, which is a relatively complex model.

Because the images take up too much memory, we can optimize the network structure and adjust the efficiency of the video memory to reduce the training time of the model. Moreover, in the field of medical image processing, there are many methods that use GAN to expanse datasets. However, by now, there is no meaningful and universal quantitative way to judge the authenticity of the composite images generated by these methods. Therefore, the improvement and application of generative adversarial networks in the field of medical image processing is a direction worthy of in-depth research.

## Conclusion

In this study, we mainly conduct a lot of research on the 3D segmentation of liver CT images, which has mainly achieved the following research results. Firstly, in view of the poor effect of 3D U-Net network feature extraction and insufficient accuracy of liver segmentation results, the SE module and pyramid pooling module are introduced into the 3D U-Net network to improve the accuracy of the segmentation results. Secondly, in view of the difficulty in obtaining labeled 3D CT images, the improved 3D U-Net network is embedded in generative adversarial network as the discriminator. In view of the poor quality of using random noise as input to generate 3D abdominal CT fake images, more real image distribution information is added to the input of the network, and a deep convolutional neural network is designed as the generator based on feature restoration method to generate more realistic fake images. Finally, the network model was applied in the 3D segmentation of kidney to test the generalization ability of the model, which have showed that the model can also obtain better segmentation results on the kidney dataset.

## Data Availability Statement

The original contributions presented in the study are included in the article/supplementary material, further inquiries can be directed to the corresponding author/s.

## Author Contributions

RH and SX conceived the study. SX performed the design and implementation of the algorithm. YY and YashuL helped the algorithm design and implementation. RH, SX, and HZ wrote the manuscript. QL commented on and approved the manuscript. NZ and YangL contributed to the segmentation of liver. All authors have read and approved the final manuscript.

## Funding

This work was supported by the Collaborative Innovation Center for Prevention and Treatment of Cardiovascular Disease of Sichuan Province (CICPTCDSP) [Grant Number xtcx2019-01] (to HZ).

## Conflict of Interest

The authors declare that the research was conducted in the absence of any commercial or financial relationships that could be construed as a potential conflict of interest.

## Publisher's Note

All claims expressed in this article are solely those of the authors and do not necessarily represent those of their affiliated organizations, or those of the publisher, the editors and the reviewers. Any product that may be evaluated in this article, or claim that may be made by its manufacturer, is not guaranteed or endorsed by the publisher.
